# A Computational Study of Astrocytic GABA Release at the Glutamatergic Synapse: EAAT-2 and GAT-3 Coupled Dynamics

**DOI:** 10.3389/fncel.2021.682460

**Published:** 2021-07-12

**Authors:** Bronac Flanagan, Liam McDaid, John Joseph Wade, Marinus Toman, KongFatt Wong-Lin, Jim Harkin

**Affiliations:** Intelligent Systems Research Centre, Ulster University, Derry, United Kingdom

**Keywords:** astrocyte, sodium-signaling, neurotransmission, synapse, glutamate, GABA

## Abstract

Neurotransmitter dynamics within neuronal synapses can be controlled by astrocytes and reflect key contributors to neuronal activity. In particular, Glutamate (Glu) released by activated neurons is predominantly removed from the synaptic space by perisynaptic astrocytic transporters EAAT-2 (GLT-1). In previous work, we showed that the time course of Glu transport is affected by ionic concentration gradients either side of the astrocytic membrane and has the propensity for influencing postsynaptic neuronal excitability. Experimental findings co-localize GABA transporters GAT-3 with EAAT-2 on the perisynaptic astrocytic membrane. While these transporters are unlikely to facilitate the uptake of synaptic GABA, this paper presents simulation results which demonstrate the coupling of EAAT-2 and GAT-3, giving rise to the ionic-dependent reversed transport of GAT-3. The resulting efflux of GABA from the astrocyte to the synaptic space reflects an important astrocytic mechanism for modulation of hyperexcitability. Key results also illustrate an astrocytic-mediated modulation of synaptic neuronal excitation by released GABA at the glutamatergic synapse.

## Introduction

Glutamate (Glu) and γ-aminobutyric acid (GABA) are the brain’s most prevalent excitatory and inhibitory neurotransmitters, respectively (Petroff, 2002). Exposure of neurons expressing appropriate excitatory ionotropic receptors, including *N*-methyl-D-aspartate receptors (NMDA-Rs) and α-amino-3-hydroxy-5-methyl-4-isoxazolepropionic acid receptors (AMPA-Rs), to Glu can result in an influx of cations elevating the neuronal membrane potential toward the firing threshold (Meldrum, 2000). Conversely, the exposure of neurons expressing GABA ionotropic receptors (GABA_A_-Rs) to GABA can result in a hyperpolarising current, decreasing the neuronal membrane potential away from the firing threshold (Sigel and Steinmann, 2012). As the metabolism of Glu and GABA is an intracellular process, these neurotransmitters must be rapidly removed from the extracellular space (ECS) by their corresponding transporters, to avoid the over-exposure of the agonist to the excitatory and inhibitory ionotropic receptors (Petroff, 2002). Broadly speaking on a network level, a balance between excitation and inhibition is necessary for normal brain activity (Fellin et al., 2006). Moreover, imbalance of excitatory/inhibitory transmission is believed to underlie such conditions as epilepsy (Clasadonte and Haydon, 2012; Coulter and Steinhäuser, 2015), autism spectrum disorders (Pizzarelli and Cherubini, 2011), schizophrenia (Coyle, 2004) and Alzheimer’s disease (Robinson, 2000). In particular, studies have indicated altered Glu and GABA concentrations within focal seizure sites (During and Spencer, 1993; Petroff, 2002), favoring excitatory neuronal behavior over inhibition.

In previous work (Flanagan et al., 2018) the effects of synaptic glutamatergic dynamics on postsynaptic firing were explored, where it was found that an increased astrocytic Glu content was sufficient to slow synaptic Glu clearance due to reduced driving force across the excitatory amino-acid transporter 2 (EAAT-2, homologue of GLT-1). Altered sodium (Na^+^) and calcium (Ca^2+^) dynamics within the astrocyte were also found and are attributed to variations in the EAAT-2 currents.

A strong extracellular-to-intracellular Na^+^ concentration gradient is imperative for a range of homeostatic functions, including neurotransmitter transport (Kirischuk et al., 2012; Verkhratsky and Nedergaard, 2018). The influx of Glu across the astrocytic membrane, against a large (∼10^6^ times) concentration gradient, by EAAT-2 requires the concerted transport of 3Na^+^ and 1H^+^ and counter-transport of 1K^+^ for each Glu ion (Zerangue and Kavanaugh, 1996). Due to its Na^+^-dependence, the reversal potential of the EAAT-2 lies well above the astrocytic resting membrane potential of ∼−80 mV (Verkhratsky and Nedergaard, 2018), ensuring the astrocytic influx of Glu upon synaptic Glu release.

In contrast, the reversal potential of GABA transporter type-3 (GAT-3) approximates to the astrocytic membrane potential and is also dependent on co-transport substrate (Na^+^ and Cl^–^) concentrations at equilibrium. Where the clearance of synaptic-released Glu appears as a predominantly astrocytic function (Danbolt, 2001), synaptic-released GABA is mostly retaken by the releasing neuron and subsequently recycled into vesicles (Hertz et al., 1999; Schousboe et al., 2014). As the GABA concentration in the ECS close to the astrocyte would be unlikely to increase based on this synaptic self-recovery of neurotransmitter, the direction of GABA transport by GAT-3 transporter is highly sensitive to fluctuations in astrocytic and extracellular ionic concentrations. In other words, the development of an astrocytic [Na^+^] microdomain (Breslin et al., 2018) may be sufficient to prompt the release of GABA into the ECS by disturbing the electrochemical potential of the transporter and eliciting an efflux of transporter substrates from the astrocyte. In particular, EAAT-2 activation has been observed experimentally to initiate the GAT-3-mediated release of GABA (Héja et al., 2012), believed to modulate tonic neuronal inhibition through the action of GABA_A_-Rs (Rossi et al., 2003; Farrant and Nusser, 2005; Héja et al., 2012). Considering this observation, the co-localization of the major Glu and GABA transporters, EAAT-2 and GAT-3, respectively, on the astrocytic membrane (Minelli et al., 1996; Héja et al., 2012; Kirischuk et al., 2012) may indicate a finely balanced excitatory-inhibitory mechanism: the uptake of Glu coupled to the astrocytic release of cytoplasmic GABA (Héja et al., 2012).

Traditionally, the classification of neuronal synapses was determined by the presynaptic neuron-released neurotransmitter (O’Rourke et al., 2012), for example, the presynaptic neuron of a glutamatergic synapse would release Glu by activity-induced exocytosis (Kandel et al., 2012). Consequently, computational models of neuron-astrocyte synapses consider neuronal and astrocytic activity as a function of neuronal-released neurotransmitter (Bentzen et al., 2009; Allam et al., 2012; Tewari and Majumdar, 2012; Li et al., 2016; Hübel et al., 2017). The neurotransmitter almost exclusively modeled at the tripartite synapse is Glu (Manninen et al., 2018), and the possibility of a secondary neurotransmitter at the same synapse has hitherto been ignored. The significance and novelty of this paper lies in the fact that it explores the astrocyte-mediated symbiosis of two neurotransmitters, Glu and GABA, at the glutamatergic synapse with a view to creating a more complete view of ionic dynamics at the tripartite synapses.

Furthermore, this paper considers the electrochemical potentials of both EAAT-2 and GAT-3 proteins with a view to (a) explain experimentally observed phenomena, (b) explore the effectiveness of this balance where astrocytic Glu concentrations are elevated and (c) predict the effects of this balance for postsynaptic neuron activity.

## Materials and Methods

**FIGURE 1:**
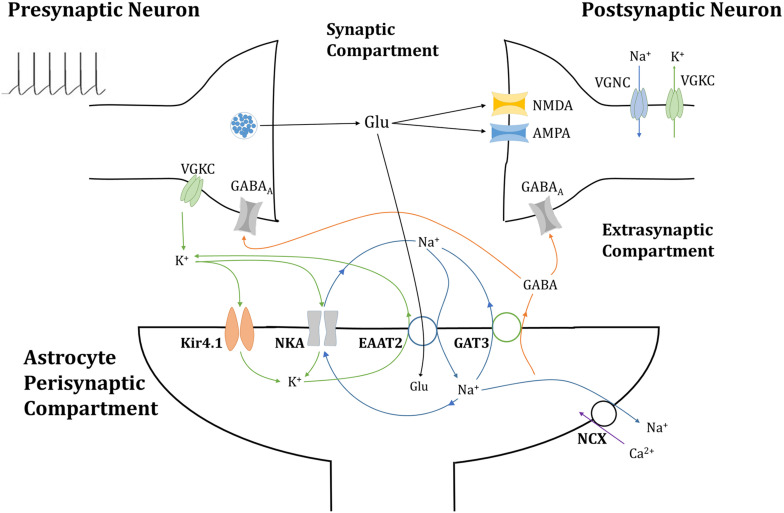
Tripartite glutamatergic synapse compartment model: consisting of neuronal, synaptic, and astrocytic compartments, within which ionic concentrations are dynamic and neuronal membrane currents respond to neurotransmitter-mediated ionotropic currents (not to scale). Glutamate (Glu) neurotransmitter is released into the synapse by the presynaptic neuron in a membrane potential-dependent mechanism, where it activates neurotransmitter-gated ionotropic receptors NMDA and AMPA channels located on the postsynaptic neuron. This postsynaptic membrane activity triggers the activation of voltage-gated sodium (Na^+^) and potassium (K^+^) channels (VGNC and VGKC, respectively), also located on the postsynaptic neuronal membrane. Synaptic Glu is exclusively taken up by astrocytic membrane-bound excitatory amino acid transporter 2 (EAAT2), along with synaptic Na^+^ and the counter-transport of astrocytic K^+^. The influx of Na^+^ into the astrocytic compartment triggers the release of GABA (and Na^+^) through GABA transporter type 3 (GAT3), into the synaptic compartment, where it activated inhibitory ionotropic GABA_A_ receptors located both on the pre- and postsynaptic neuronal membranes. Synaptic K^+^ transport is completed with the inclusion of inwardly rectifying K^+^ channels (Kir4.1) and the Na^+^/K^+^ ATPase transporter (NKA). Na^+^ dynamics are completed by the NKA and reversible Na^+^/Ca^2+^ exchanger (NCX).

To consider the effects of synaptic neurotransmitter fluxes, namely the presynaptic and postsynaptic neuronal synaptic-driven dynamics, the model in Figure 1 was considered. The model extends a previously developed framework (Flanagan et al., 2018) to characterize tripartite synapse signaling, to include GABAergic signaling as well. A pulsed depolarizing current of 5 μA/cm^2^ was applied for 50 s to the presynaptic neuronal membrane, generating a presynaptic neuronal 10 Hz firing rate. Consideration was also given to the influence of astrocytic Glu content, in line with previous simulation (Flanagan et al., 2018), specifically in the transport of Glu across the astrocytic membrane. To achieve this, the basal astrocytic [Glu] of 1.5 mM and 5 mM were chosen to represent the bounds of the physiological range (Attwell et al., 1993) and the basal astrocytic [Glu] of 10 mM hypothesized pathological state (Flanagan et al., 2018), following glutamine synthetase downregulation (Eid et al., 2008; Perez et al., 2012). At each neuronal spike, Glu and K^+^ were released from the presynaptic neuron into the synaptic cleft. The model was also simulated with the exclusion of GAT-3 to act as a control in determining the role of EAAT-2-induced GAT-3 transport at the neuronal synapse. The key ions considered were Na^+^, K^+^, Glu, Ca^2+^ and GABA.

### Presynaptic Membrane and Neurotransmitter Dynamics

The presynaptic neuron is modeled using a Hodgkin-Huxley-based (Hodgkin and Huxley, 1952; Golomb et al., 2006) description for voltage-gated Na^+^ and K^+^ dynamics. The presynaptic membrane potential uses the formalism.

(1) $${\text{C}}_{\text{M}}\frac{{\text{dV}}_{\text{m},\text{pre}}}{\text{dt}}=-({\text{I}}_{\text{Na},\text{Preneuron}}+{\text{I}}_{\text{K},\text{Preneuron}}+{\text{I}}_{\text{L},\text{Preneuron}}+{\text{I}}_{\text{PreGABAA}}+{\text{I}}_{\text{app}})$$

where I_Na,Preneuron_, I_K,Preneuron_ and I_L__,Preneuron_ reflect voltage-gated Na^+^, K^+^ and leak presynaptic currents, respectively, and are described in Table 1 with parameters enumerated in Table 2. I_PreGABAA_ is the GABA_A_ mediated current in response to synaptic astrocyte-released GABA, see below, and I_app_ is an applied stimulus.

**TABLE 1:** Neuron membrane dynamics.

Membrane currents	Description	Equation(s)	Source
_*Na*,*neuron*_	Voltage-gated Na^+^ current	$I_{N\,a,n\,e\,u\,r\,o\,n}=g_{N\,a,n\,e\,u\,r\,o\,n}\,m_{\infty }^{3}\,h\,\left(V_{m}-E_{N\,a}\right)$	Golomb et al., 2006
		$E_{\mathrm{Na}}=\frac{\mathrm{RT}}{F}\,\ln\,\left(\frac{{\mathrm{Na}}_{\mathrm{syn}}}{{\mathrm{Na}}_{\mathrm{neuron}}}\right)$	
		$m_{\infty }={\left(1+e^{\left(-\frac{V_{m}-\left(-30\right)}{9.5}\right)}\right)}^{-1}$	
		$\frac{\mathrm{dh}}{\mathrm{dt}}=\frac{h_{\infty }-h}{\tau_{h}}$	
		$\tau_{h}=\left(0.1+\frac{0.75}{1+e^{\left(-\frac{V_{m}-\left(-40.5\right)}{-6}\right)}}\right)$	
		$h_{\infty }={\left(1+e^{\left(-\frac{V_{m}-\left(-45\right)}{-7}\right)}\right)}^{-1}$	
I_K,*neuron*_	Voltage-gated K^+^ current	I_K,*neuron*_=g_k,*neuron*_n^4^(V_m_−E_K_)	Golomb et al., 2006
		$E_{K}=\frac{\mathrm{RT}}{F}\,\ln\,\left(\frac{K_{\mathrm{syn}}}{K_{\mathrm{neuron}}}\right)$	
		$\frac{\mathrm{dn}}{\mathrm{dt}}=\frac{n_{\infty }-n}{\tau_{n}}$	
		$\tau_{n}=0.1+\frac{0.5}{1+e^{\left(-\frac{V_{m}-\left(-27\right)}{-15}\right)}}$	
		$n_{\infty }={\left(1+e^{-\frac{V_{m}-\left(-35\right)}{10}}\right)}^{-1}$	
I_L,N_	Membrane leak current	I_L,N_=g_L_(V_m_−E_L_)	Golomb et al., 2006
I_*NMDA*_	NMDA-mediated current	I_*NMDA*_=g_*NMDA*_r_*NMDA*_(V_m_−E_*NMDA*_)*Mg*_V_	Destexhe et al., 1998
		$\frac{{\mathrm{dr}}_{\mathrm{NMDA}}}{\mathrm{dt}}=\alpha_{N\,M\,D\,A}\,\left[\text{Glu}\right]\,\left(1-r_{\mathrm{NMDA}}\right)-\beta_{\mathrm{NMDA}}\,r_{\mathrm{NMDA}}$	
		$M\,g_{V}={\left(1+e^{\frac{-0.062\,V_{m}\,M\,G}{3.57}}\right)}^{-1}$	
I_*AMPA*_	AMPA-mediated current	I_*AMPA*_=g_*AMPA*_r_*AMPA*_(V_m_−E_*AMPA*_)	Destexhe et al., 1998
		$\frac{{\mathrm{dr}}_{A\,\text{MPA}}}{\mathrm{dt}}=\alpha_{\mathrm{AMPA}}\,\left[\text{Glu}\right]\,\left(1-r_{\mathrm{AMPA}}\right)-\beta_{\mathrm{AMPA}}\,r_{\mathrm{AMPA}}$	
I_*GABAA*_	GABA_A_-mediated current	I_*GABAA*_=g_*GABAA*_r_*GABAA*_(V_m_−E_*GABAA*_)	Destexhe et al., 1998
		$\frac{{\mathrm{dr}}_{\mathrm{GABAA}}}{\mathrm{dt}}=\alpha_{\mathrm{GAB}\,A\,A}\,\left[\text{GABA}\right]\,\left(1-r_{\mathrm{GABAA}}\right)-\beta_{\mathrm{GABAA}}\,r_{\mathrm{GABAA}}$	

**TABLE 2:** Neuronal membrane model parameters.

Parameter	Description	Value	Units	Source
*V*n_*eq*_	Resting postsynaptic neuron membrane potential	–71	*mV*	Calculated at equilibrium values
g_*Nan*_	Neuronal voltage-gated Na^+^ channel conductance	35	*mSc*m^−2^	Hodgkin and Huxley, 1952
g_*kDr*_	Neuronal voltage-gated K^+^ channel conductance	6	*mSc*m^−2^	Hodgkin and Huxley, 1952
C_m_	Neuron capacitance	1	μ*Fc*m^−2^	Hodgkin and Huxley, 1952
g_L_	Neuronal leak channel conductance	0.0112	*mSc*m^−2^	Hodgkin and Huxley, 1952
E_L_	Neuron leak conductance	–74.6	*mV*	Calculated
g_*NMDA*_	Synaptic NMDA-R maximal conductance	0.026	*mSc*m^−2^	Destexhe et al., 1998
g_*AMPA*_	Synaptic AMPA-R maximal conductance	0.0145	*mSc*m^−2^	Destexhe et al., 1998
g_*GABAA*_	Synaptic GABAA-R maximal conductance	0.0145	*mScm*^−2^	Destexhe et al., 1998
E_*GABAA*_	GABA_A_ reversal potential	–85	*mV*	Destexhe et al., 1998
E_*AMPA*_	AMPA reversal potential	0	*mV*	Destexhe et al., 1998
E_*NMDA*_	GABA_A_ reversal potential	0	*mV*	Destexhe et al., 1998
α_*GABAA*_	GABA_A_ forward rate constant	5×10^2^	M^−1^*msec*^−1^	Destexhe et al., 1998
α_*AMPA*_	AMPA forward rate constant	1.1×10^3^	M^−1^*msec*^−1^	Destexhe et al., 1998
α_*NMDA*_	NMDA forward rate constant	72	M^−1^*msec*^−1^	Destexhe et al., 1998
β_*GABAA*_	GABA_A_ backward rate constant	0.72	*msec*^−1^	Destexhe et al., 1998
β_*AMPA*_	AMPA backward rate constant	0.190	*msec*^−1^	Destexhe et al., 1998
β_*NMDA*_	NMDA backward rate constant	6.6×10^−3^	*msec*^−1^	Destexhe et al., 1998

To improve upon previous work (Flanagan et al., 2018), a more realistic description of synaptic resources was accounted for to preserve biological realism. To this end, the model adopts the Tsodyks-Markram model (Tsodyks et al., 1998) description for the use of synaptic resources within a facilitating synapse, so that any alteration of the neuronal activity would be directly influenced by the GABA-induced inhibition rather than the nature of the synapse model. This model describes the fraction of recovered resources (x), active resources (y) and inactive resources (z) using the model scheme

(2) $$\frac{\text{dx}}{\text{dt}}=\frac{\text{z}}{\tau_{\text{r}}}-\text{Ux}\,\delta \,\left(\text{t}-{\text{t}}_{\text{sp}}\right)$$

(3) $$\frac{\text{dy}}{\text{dt}}=-\frac{\text{y}}{\tau_{\text{i}}}+\text{Ux}\,\delta \,\left(\text{t}-{\text{t}}_{\text{sp}}\right)$$

(4) $$\frac{\text{dz}}{\text{dt}}=\frac{\text{y}}{\tau_{\text{i}}}-\frac{\text{z}}{\tau_{\text{r}}}$$

using parameters detailed in Table 3.

**TABLE 3:** Presynaptic resource model parameters.

Parameter	Description	Value	Units	Source
τ_i_	Synaptic inactivity time constant	0.003	*sec*	Tsodyks et al., 1998
τ_r_	Synaptic recovery time constant	0.800	*sec*	Tsodyks et al., 1998
*U*	Synaptic efficacy utilization fraction	0.5	∼	Tsodyks et al., 1998

In this model the amount of Glu released by the presynaptic neuron is proposed to be proportional to the fraction of active resources (Eq. 3) is scaled by constant parameter of 0.1 mM, chosen to sufficiently perturb the system under all cases, namely to excite the postsynaptic membrane to its firing threshold. At each presynaptic neuronal spike, Glu is released by the presynaptic neuron into the synaptic compartment, along with a small amount of K^+^ representing the input to the system.

### Astrocytic Membrane Dynamics

Astrocytic membrane ionic currents, subject to changes in ionic concentrations (Glu, K^+^, Na^+^, Ca^2+^ and GABA), are then calculated using the following equations

(5) $${\text{I}}_{\text{Na},\text{ast}}=1.5\,{\text{I}}_{\text{EAAT}}+3\,{\text{I}}_{\text{NKA}}+3\,{\text{I}}_{\text{NCX}}+2\,I_{\text{GAT}}+{\text{I}}_{\text{Na},\text{L}}$$

(6) $${\text{I}}_{\text{K},\text{ast}}=-0.5\,{\text{I}}_{\text{EAAT}}+2\,{\text{I}}_{\text{NKA}}+{\text{I}}_{\text{Kir}}+{\text{I}}_{\text{K},\text{L}}$$

(7) $${\text{I}}_{\text{Glu},\text{ast}}=-0.5\,{\text{I}}_{\text{EAAT}}+{\text{I}}_{\text{Glu},\text{L}}$$

(8) $${\text{I}}_{\text{Ca},\text{ast}}=-2\,{\text{I}}_{\text{NCX}}+{\text{I}}_{\text{Ca},\text{L}}$$

(9) $${\text{I}}_{\text{GABA},\text{ast}}={\text{I}}_{\text{GAT}}$$

within this scheme I_EAAT_, I_NKA_, I_NCX_, I_GAT_ and I_Kir_ denote EAAT-2, NKA, Na^+^-Ca^2+^ exchanger (NCX), GAT-3 and inwardly rectifying K^+^ channel (Kir_4.1_) generated currents, respectively. The inclusion of Na^+^, K^+^, Glu, and Ca^2+^ leak currents, denoted I_Na,__L_, I_K,__L_, I_Glu,__L_, and I_Ca__L, L_ respectively, provide model stability. Each transport current is calculated using the existing concentration of its corresponding substrate(s), the equations of which are found in Table 4 and parameters used in the model are contained in Table 5. To study the effects of these currents on concentrations alone, the membrane potential of the astrocyte is set to be constant.

**TABLE 4:** Astrocyte membrane transporter equations.

Membrane current	Equation(s)	Source
I_*Kir*_	$I_{\mathrm{Kir}}=g_{\mathrm{kir}}*\sqrt{K_{\mathrm{syn}}}*\left(V_{a}-E_{K}\right);$	Witthoft et al., 2013
I_*NKCC*_	$I_{\mathrm{NKCC}}=I_{\mathrm{NKCC},\max}\,l\,o\,g\,\left(\left(\frac{{\mathrm{Na}}_{\mathrm{syn}}}{{\mathrm{Na}}_{\mathrm{ast}}}\right)\,\left(\frac{K_{\mathrm{syn}}}{K_{\mathrm{ast}}}\right)\,{\left(\frac{{\mathrm{Cl}}_{\mathrm{syn}}}{{\mathrm{Cl}}_{\mathrm{ast}}}\right)}^{2}\right)$	Witthoft et al., 2013
I_*NCX*_	$I_{\mathrm{NCX}}=I_{\mathrm{NCXmax}}\,\left({\left(\frac{{\mathrm{Na}}_{\mathrm{ast}}}{{\mathrm{Na}}_{\mathrm{syn}}}\right)}^{3}\,e^{\frac{\gamma \,{\mathrm{FV}}_{a}}{\mathrm{RT}}}-\left(\frac{{\mathrm{Ca}}_{\mathrm{ast}}}{{\mathrm{Ca}}_{\mathrm{syn}}}\right)\,e^{\frac{\left(\gamma -1\right)\,{\mathrm{FV}}_{a}}{\mathrm{RT}}}\right)$	Schutter and Smolen, 1998
I_*EAAT*_	I_*EAAT*_=−α_*EAAT*_⋅e^−β_*EAAT*_(V_a_−E_*EAAT*_)^	
	$E_{\mathrm{EAAT}}=\frac{\mathrm{RT}}{2\,F}\,\ln\,\left({\left(\frac{{\mathrm{Na}}_{\mathrm{syn}}}{{\mathrm{Na}}_{\mathrm{ast}}}\right)}^{3}\,\left(\frac{H_{\mathrm{syn}}}{H_{\mathrm{ast}}}\right)\,\left(\frac{{\mathrm{Glu}}_{\mathrm{syn}}}{{\mathrm{Glu}}_{\mathrm{ast}}}\right)\,\left(\frac{K_{\mathrm{ast}}}{K_{\mathrm{syn}}}\right)\right)$	Flanagan et al., 2018
I_*NKA*_	$I_{\mathrm{NKA}}=I_{\mathrm{NKAmax}}\,\left(\frac{{\mathrm{Na}}_{\mathrm{ast}}^{1.5}}{{\mathrm{Na}}_{\mathrm{ast}}^{1.5}+K_{\mathrm{Nai}}^{1.5}}\right)\,\left(\frac{K_{\mathrm{syn}}}{K_{\mathrm{syn}}+K_{\mathrm{Ke}}}\right)$	Halnes et al., 2013
I_*GAT*_	I_*GAT*_=g_*gat*_(*V*__*a*_−E_*GAT*_);	
	E_*GAT*_ = $\frac{\mathrm{RT}}{F}\,\ln\,\left({\left(\frac{{\mathrm{Na}}_{\mathrm{syn}}}{{\mathrm{Na}}_{\mathrm{ast}}}\right)}^{2}\,\left(\frac{{\mathrm{GABA}}_{\mathrm{syn}}}{{\mathrm{GABA}}_{\mathrm{ast}}}\right)\,\left(\frac{{\mathrm{Cl}}_{\mathrm{syn}}}{{\mathrm{Cl}}_{\mathrm{ast}}}\right)\right)$	Adapted from Verkhratsky and Nedergaard (2018)
I_X,L_	I_X,L_=g_X_(V_a_−E_X_)	
	$E_{X}=\frac{\mathrm{RT}}{z_{X}\,F}\,l\,n\,\left(\frac{X_{\mathrm{out}}}{X_{\mathrm{in}}}\right)\,f\,o\,r\,X=N\,a_{\mathrm{ast}},K_{\mathrm{ast}},G\,l\,u_{\mathrm{ast}},C\,a_{\mathrm{ast}}$	

**TABLE 5:** Astrocytic membrane transporter parameters.

Parameter	Description	Value	Units	Source
F	Faraday’s constant	96480	C mol^−1^	
R	Ideal gas constant	8.3145	J K^−1^ mol^−1^	
T	Temperature	310	K	
*Vol*_S_	Synaptic Volume	8.5883 × 10^−16^	L	Breslin et al., 2018
*Vol*_A_	Astrocytic Volume	1.885 × 10^−17^	L	Breslin et al., 2018
*S*_A_	Astrocytic membrane surface area	1.4137 × 10^−13^	*m*^2^	Breslin et al., 2018
*P*_NKAmax_	Maximal NKA current	0.1081	A m^−^2	Adapted from Halnes et al. (2013)
K_*Nai*_	NKA affinity for Na^+^	1.5	mM	Halnes et al., 2013
K_*Ke*_	NKA affinity for K^+^	10	mM	Halnes et al., 2013
I_*NCXmax*_	NCX max current density	0.01	A m^−2^	Schutter and Smolen, 1998
γ	NCX partition parameter	0.5	[]	Schutter and Smolen, 1998
α_*EAAT*_	EAAT scaling constant	2 = 10^−4^	A m^−2^	Flanagan et al., 2018
β_*EAAT*_	EAAT scaling constant	29.2	V^−1^	Flanagan et al., 2018
g_*kir*_	K^+^ conductance	1440	S m^−2^	Adapted from Witthoft et al. (2013)
E_*Kir*_	Reversal potential for Kir4.1	0.025	V	Witthoft et al., 2013
g_*gat*_	GAT3 conductance	2.1 × 10^2^	*S*m^−2^	Maximized parameter
z_*Na*_	Na^+^ valency	+1	[]	
z_K_	K^+^ valency	+1	[]	
z_*Ca*_	Ca^2+^ valency	+2	[]	
z_*Glu*_	Glu valency	−1	[]	

Currents are converted to ionic fluxes by Faraday’s law, where the change in the astrocytic concentration of ion X is given by

$$\frac{{\text{dX}}_{\mathrm{ast}}}{\mathrm{dt}}=-\frac{{\text{I}}_{X,\mathrm{ast}}}{\mathrm{zF}}\,S_{A}\,\text{Vo}\,l_{A}$$

(10) $$\left\{X={\mathrm{Na}}^{+},K^{+},\mathrm{Glu},{\mathrm{Ca}}^{2+},\mathrm{GABA}\right\}$$

and corresponding change in synaptic concentration given by

$$\frac{{\text{dX}}_{\mathrm{syn}}}{\mathrm{dt}}=\frac{{\text{I}}_{X,\mathrm{ast}}}{\mathrm{zF}}\,S_{A}\,\text{Vo}\,l_{S}$$

(11) $$\left\{X={\mathrm{Na}}^{+},K^{+},\mathrm{Glu},{\mathrm{Ca}}^{2+},\mathrm{GABA}\right\}$$

using the surface area of the peri-synaptic astrocytic membrane (S_A_) and volume of astrocyte (Vol_A_) and synaptic compartments (Vol_S_) as parameters.

### Postsynaptic Membrane Dynamics

Synaptic Glu and GABA concentrations are also used to calculated local postsynaptic neuronal membrane dynamics, the neurotransmitters activating corresponding receptors on the postsynaptic terminal, the equations for which are found in Table 1 and the parameters used in Table 2.

The localized effect of these neurotransmitter-driven currents on the postsynaptic neuron are also calculated using a Hodgkin-Huxley-based (Hodgkin and Huxley, 1952; Golomb et al., 2006) description, with the change in the postsynaptic neuron membrane potential (V_m_) is thus calculated as the (negative) sum of intrinsic voltage-gated Na^+^ and K^+^ currents, a leak current and synaptic NMDA, AMPA and GABA_A_ mediated currents (Destexhe et al., 1998) as given by

(12) $$C_{M}\frac{{\mathrm{dV}}_{m}}{\mathrm{dt}}=-(I_{\mathrm{Na},\mathrm{neuron}}+I_{K,\mathrm{neuron}}+I_{L,\mathrm{neuron}}+I_{\mathrm{NMDA}}+I_{\mathrm{AMPA}}+I_{\mathrm{GABAA}}).$$

### Model Simulation

The simulation uses the forward Euler numerical integration scheme with 0.01 ms time step using MATLAB R2017b. Each time step of the model was considered in three separate settings, where only the initial astrocytic Glu concentration differs, i.e., [Glu]_ast,eq_ = 1.5, 5, and 10 mM and leak conductances are adjusted accordingly so that equilibrium conditions are met initially.

## Results

Results are split into two sections: the first considers the resulting ionic changes due to the simulation while the second considers the effects of the neurotransmitter dynamics on the pre- and postsynaptic neuronal membrane. As astrocytes are considered the main controller of ionic homeostasis, this model only considers changes in ionic concentrations due to astrocytic membrane-mediated currents.

### Astrocyte-Mediated Neurotransmitter and Ionic Dynamics

#### EAAT Activation Leads to Increase of Astrocytic [Na^+^]

**FIGURE 2:**
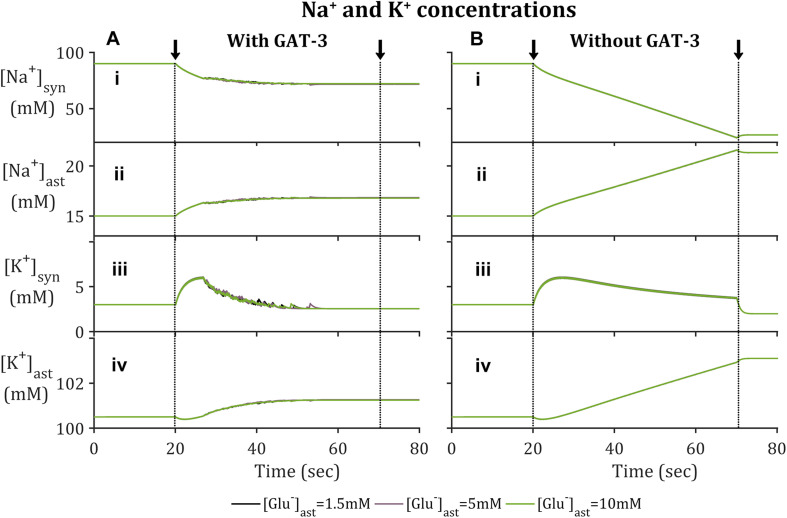
Astrocytic and synaptic concentrations of Na^+^ and K^+^ where the model **(A)** includes GAT-3 activity and **(B)** where GAT-3 activity is not included; **(i–iv)** describes (respectively) synaptic Na^+^, astrocytic [Na^+^], synaptic [K^+^] and astrocytic [K^+^]. The results show the ionic concentrations of a simulation in which the presynaptic neuron is subject to an applied current, pulsed at a frequency of 10 Hz over a 50 s window starting at 20 s (window of applied current given by arrows and dashed vertical lines).

In agreement with previously reported findings (Breslin et al., 2018; Flanagan et al., 2018), the activation of EAAT-2 transporter by neuronal-released Glu was sufficient to generate an astrocytic influx of Na^+^ within the model containing GAT-3 (Figure 2A). This resulted in a decrease in synaptic [Na^+^] (Figure 2A.i) and a corresponding increase in astrocytic [Na^+^] (Figure 2A.ii). The simulation was repeated without GAT-3, as a control, and found similar, yet exaggerated results (Figures 2B.i,ii). This is expected from the dual effect of decreased EAAT-2-mediated influx, resulting from reduced presynaptic neuronal activity, and the Na^+^-dependent efflux of Na^+^ through GAT-3. [K^+^] increased in the synaptic compartment (Figures 2A.iii,B.iii) with neuronal activity and correspondingly decreased in the astrocytic compartment (Figures 2A.iv,B.iv). Considering this inverse behavior between intracellular and extracellular concentration change, these results suggest that the astrocytic efflux of K^+^ by EAAT-2 dominates over the influx of K^+^ by the Na^+^/K^+^ ATPase (NKA), with a net increase of [K^+^] in the cleft. However, the rate of change of [K^+^] in both compartments was higher where the model did not include GAT-3 (Figures 2B.iii,iv) because the NKA is sensitive to astrocytic [Na^+^] and therefore in the absence of GAT-3 a heightened NKA activity results. This generated a more pronounced change in [K^+^] in both compartments. Due to the fact this is not a closed system, as K^+^ has been injected due to the presynaptic activity, the concentration levels do not return to baseline following the simulation.

#### EAAT-Mediated [Na_ast_] Increase Is Sufficient to Reverse GAT-3

The reversal potential of GAT-3 (E_GAT_) is heavily dependent on the [Na^+^] gradient across the astrocytic membrane and, at equilibrium conditions, is close to parity with the astrocytic membrane potential (Verkhratsky and Nedergaard, 2018). This indicates that the direction of its mediated ionic fluxes is highly sensitive to any change in [Na^+^]. The reversible nature of the transporter is demonstrated in Figure 4B, where a reduction in the transmembrane [Na^+^] gradient is sufficient to reduce E_GAT_ to below the astrocytic membrane potential, facilitating the release of its substrates, GABA, Na^+^ and Cl^–^ (not included in this model). Note that the recovery rate of E_GAT_ following activation is markedly faster where astrocytic Glu is lower, which can be attributed to the correlation between EAAT-2 activity and astrocytic [Glu] (Flanagan et al., 2018). EAAT-2 activity increased as astrocytic Glu decreased and thus EAAT-2- mediated influx of Na^+^ resulted in heightened NKA activity. This increase in transmembrane currents resulted in a faster recovery of E_GAT_.

As expected, the inclusion of a GAT-3 transporter restricted astrocytic [Na^+^] as the concentration dependent GAT-3 reversal potential dropped below the astrocytic membrane potential, resulting in the net efflux of Na^+^ through this transporter. Little difference was recorded in comparison between simulation setups regarding basal astrocytic [Glu], due to the relative size of the Glu-mediated fluxes compared to the magnitude of the ionic concentrations.

#### Time Course of Synaptic Glutamate Affected by GAT-3 Activity

**FIGURE 3:**
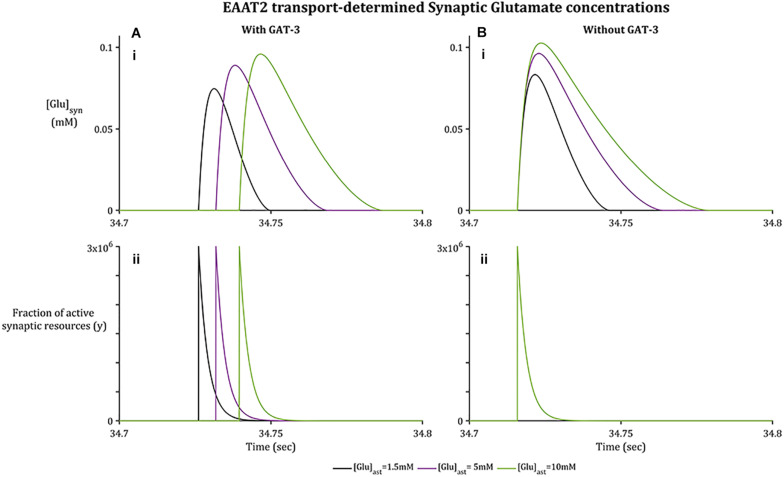
Synaptic [Glu] dynamics for a 0.1 s window within the simulation associated with basal [Glu]_ast_.**(A.i)** shows [Glu]_syn_ increasing to a higher concentration where the basal [Glu]_ast_ is higher, despite the fraction of active synaptic resources **(A.ii)** being identical in all cases. Within **(B.i)**, [Glu]_syn_ increases to a higher concentration again for all [Glu]_ast_ where the GAT3-mediated transport is not included, again despite an identical fraction of active resources **(B.ii)** being released by the presynaptic neuron.

As with GAT-3 transport, the rate of Glu transport by astrocytic EAAT-2 is largely dependent on the transmembrane [Na^+^] gradient (Zerangue and Kavanaugh, 1996; Levy et al., 1998) in addition to the Glu concentration gradient across the astrocytic membrane (Flanagan et al., 2018). In support of previously presented results (Flanagan et al., 2018), increased astrocytic Glu content promotes a longer rate of clearance and higher concentration attainment of synaptic Glu (Figure 3A.i), despite the fraction of active synaptic resources (Figure 3A.ii) being identical in all cases. This rate of clearance is increased further if GAT-3 is not included (Figure 3B) as a result of the heightened shift in [Na^+^] and [K^+^] transmembrane gradients (Figure 2).

**FIGURE 4:**
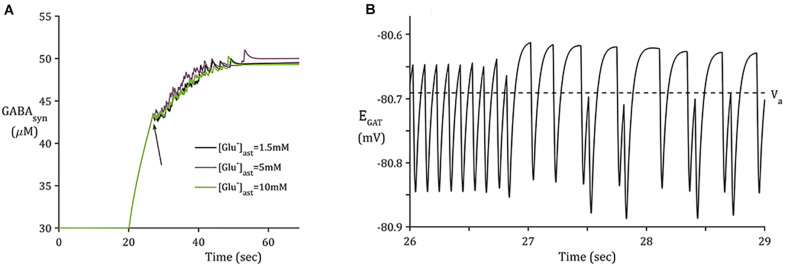
Synaptic GABA dynamics: **(A)** GABA concentration due to GAT-3 activity **(B)** 3-s window of GAT-3 reversal potential for [Glu]_ast_ = 1.5 mM (initial time of window indicated by arrow in panel **(A).**

#### Time-Scale of GAT-3-Mediated GABA Release Appropriate for Tonic Inhibition

**FIGURE 5:**
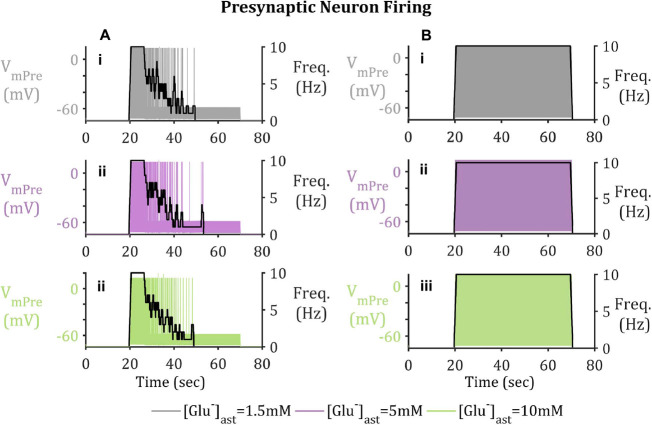
Presynaptic Neuron Membrane Activity **(A)** with and **(B)** without GAT-3-mediated GABA release. Upon activation of the presynaptic neuron by means of a pulsed applied current, Glu released into the synapse triggers the activation of EAAT-2 currents, located on the astrocytic membrane. EAAT-2 transports both Glu and Na^+^ into the astrocyte disturbing the equilibrium state of GAT-3, resulting in the release of GABA back to the presynaptic neuron, where upon binding to presynaptic membrane-bound GABA_A_-Rs inhibits the neuron, even in the presence of a pulsed applied current. **(i–iii)** shows presynaptic neuron membrane potential, overlaid with presynaptic neuron firing frequency (given in black with the right-hand y-axis). Results describe basal **(i)** [Glu]_ast_ = 1.5 mM, (**ii**) [Glu]_ast_ = 5 mM, and **(iii)** [Glu]_ast_ = 10 mM.

In contrast to the sharp increase of [Glu] (Figure 3A), as implicitly described by neuronal exocytosis, the rate of GABA release by reversed GAT-3 transport is much slower (Figure 4), increasing in line with neuronal activity and decreasing slowly as the reversal potential increases above the astrocytic membrane potential. The slow time course of GAT-3 mediated GABA release describes the tonic inhibition described by Rossi et al. (2003); Farrant and Nusser (2005), and Héja et al. (2012). As GAT-3 is predominantly controlled by [Na^+^] gradients, and from Figures 2A.i,ii, 4, it can be seen than these differ little due to basal astrocytic [Glu], little difference can be seen in the GAT-3-mediated synaptic [GABA] (Figure 4A). Note that the flux of GABA fluctuates a little in line with astrocytic [Na^+^], at ∼26 s in the simulation these fluctuations become more pronounced due to the slowing of presynaptic neuronal activity (Figure 5) as the GAT-3-mediated efflux of Na^+^ attempts to correct the equilibrium concentration gradient (Figure 4B). Following the termination of presynaptic firing and Glu-mediated ionic currents, the transmembrane [Na^+^] gradient stabilizes (Figures 2A.i,ii), resulting in no net release of GABA (Figure 4A).

### Pre- and Postsynaptic Neuron Membrane Dynamics

#### Astrocyte-Released GABA Sufficient to Suppress Presynaptic Neuronal Firing

In order to model the longer-term neuronal effects of EAAT-2-GAT-3 coupling, a similar simulation to a previously published study (Flanagan et al., 2018) was performed. The major differences between the former and latter models being the inclusion of GAT-3 transport and more realistic presynaptic firing activity. The presynaptic neuronal membrane dynamics were modeled using a Hodgkin-Huxley formalism. Within this model a pulsed periodic current of 5 μA/cm^2^ was applied, sufficient to initiate a 10 Hz presynaptic neuronal firing for 50 s. In addition to the applied current, the presynaptic neuron is exposed to inhibitory currents mediated by synaptic GABA-activating GABA_A_ receptors.

From Figure 5, the current applied to the presynaptic neuron results in an initial firing frequency of ∼10 Hz. Where synaptic GABA is released by the astrocytic GAT-3 (Figure 4) and subsequently activates presynaptic GABA_A_-Rs; GABA_A_-mediated currents then compete with the simulated applied current to generate subthreshold presynaptic potentials (Figure 5A.i), reducing the presynaptic firing frequency (Figure 5B.i). In contrast, if no GABA is released by astrocytic GAT-3, the presynaptic neuronal firing persists (Figure 5.ii).

#### Postsynaptic Neuronal GABA_A_-Receptor Activation Mediates Reduction in Hyperexcitability

**FIGURE 6:**
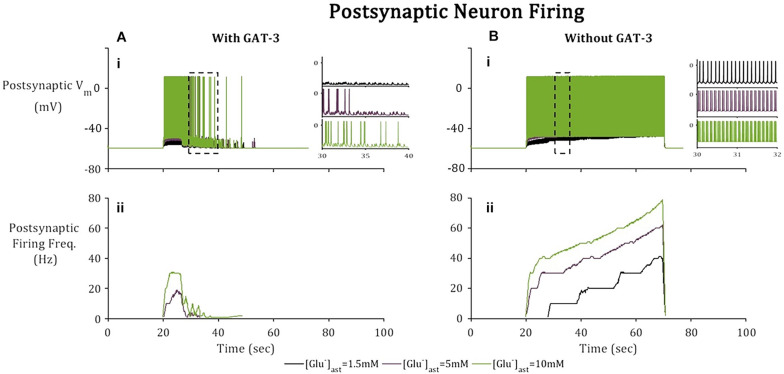
Postsynaptic neuronal activity in model simulation across 3 paradigms, [Glu]_ast_ = 1.5, 5, and 10 mM. Postsynaptic neuronal membrane is subject to **(A)** NMDA, AMPA, and GABA_A_ mediated currents with inclusion of astrocytic GAT-3-mediated GABA transport and **(B)** where the GAT-3-mediated GABA release has been omitted. **(i)** Postsynaptic neuron membrane potential and **(ii)** firing frequency as a result of an applied current (pulsed at a frequency of 10 Hz over a 50 s window starting at 20 s). All graphs depict firing frequency calculated using a moving average using window size 1 s, 10% overlap). Inset Figs describe 10 s window (left) for each of the basal [Glu]_ast_ and 2 s window (right) for each basal [Glu]_ast_, where the time window was selected according to extent of neuronal activity.

Besides GABA_A_ mediated currents, the postsynaptic terminal is subject to Glu-mediated activation of NMDA and AMPA receptors. Higher frequency firing was observed in the postsynaptic neuron (Figures 6B.i,ii) where the model does not include GAT-3 activity in comparison to the model containing GAT-3 activity (Figures 6A.i,ii). We attribute this to the dual effect of a prolonged synaptic Glu time course and over-activation of NMDA and AMPA-Rs (Flanagan et al., 2018), coupled with the exclusion of GABA-mediated inhibitory currents. Postsynaptic neuronal excitability has been shown to be correlated to time course of synaptic Glu (Flanagan et al., 2018) through activation of ionotropic NMDA and AMPA-Rs. Thus, as basal astrocytic [Glu] increased, time course of synaptic Glu increased (Figure 4) increasing the postsynaptic firing frequency (Figure 6) in both cases (with and without GAT-3).

**FIGURE 7:**
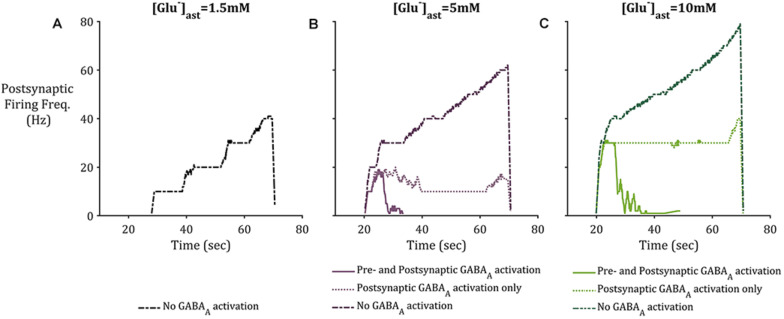
Postsynaptic neuronal firing frequency where **(A)** basal [Glu]_ast_ = 1.5 mM, **(B)** basal [Glu]_ast_ = 5 mM, and **(C)** basal [Glu]_ast_ = 10 mM. Within each plot results from the modeling simulation described above were recorded, where inclusion of astrocytic GAT-3 resulted in GABA-mediated activation of GABA_A_-Rs are either located on both presynaptic and postsynaptic neuronal membrane (*solid* line), or the postsynaptic neuronal membrane only (*dotted* line). Exclusion of GAT-3 and thus no GABA_A_-R mediated currents are included as control (*dash-dotted* line). In all cases, the inclusion of GABA dynamics resulted in diminished postsynaptic activity, specifically where [Glu]ast = 1.5 mM, which resulted in no postsynaptic activity where GABA_A_-R activation was considered.

To consider the effects of astrocytic GAT-3 mediated release of GABA for postsynaptic neuronal activity, three cases were considered; (i) where GABA activates GABA_A_-mediated inhibitory currents located on both pre- and postsynaptic neuronal membranes (as above) (ii) where GABA activates GABA_A_ mediated inhibitory currents located on the postsynaptic neuronal membrane alone and (iii) where GAT-3 is not included in the model, as a control. Figure 7 illustrates the simulation results using the setup described for each of the basal astrocytic [Glu] of 1.5, 5, and 10 mM. Where astrocytic [Glu] is low, the inclusion of GABA dynamics results in sub- threshold postsynaptic potentials only and thus the firing is completely suppressed (Figure 7A) in comparison to where GAT-3 is excluded from the model and thus GABA dynamics and GABA-mediated inhibition are ignored.

In the cases of higher astrocytic [Glu], the inclusion of GABA dynamics in both cases (i) and (ii) is sufficient to not only significantly reduce the firing frequency of the postsynaptic neuron, but also curtail the runaway Glu-mediated excitation of the neuronal membrane, as seen in the dash-dotted lines in Figures 7B,C.

## Discussion

The effects of astrocytic function and dysfunction on synaptic activity are widely researched areas in both experimental and computational fields. In this study, we focused on one particular recognized function of astrocytes: the control of extracellular Glu and GABA neurotransmitter concentrations by astrocytic transporters.

The experimental observation that astrocytic GABA transporters, GAT-3, respond to Glu mediated EAAT-2 activation (Héja et al., 2009) appears to indicate a synaptic feedback determined by the influx of shared substrate, Na^+^. Governed by the reversal potential of GAT-3, this activation mediates the release of GABA from the astrocyte and may modulate a long-lasting tonic inhibition of nearby neurons (Farrant and Nusser, 2005; Héja et al., 2009; Kersanté et al., 2013) as opposed to transient, or phasic, inhibition typically resulting from exocytotic release (Farrant and Nusser, 2005). A model to describe the interaction between EAAT-2 and GAT-3 activity was developed, exploring their effects for synaptic Glu and GABA concentrations and consequential perturbation of both the pre- and postsynaptic neuronal membrane potential.

Within this paper, a simulation sought to explore and explain the interplay of EAAT-2 and GAT-3 transport. Within the simulated time, it was noted that astrocytic GAT-3 released inhibitory neurotransmitter GABA in addition to acting as a non-ATP dependent mechanism for regulating intracellular Na^+^: a role traditionally assigned to the NKA which relies directly on ATP availability. Based on the findings of this paper, the proposition of previous experimental study (Héja et al., 2012) is supported, that GAT-3 acts to provide a modulatory effect when faced with excessive synaptic excitation. In addition, the modulatory effect is diminished where astrocytic [Glu] is elevated, as the time course of synaptic Glu is prolonged (Flanagan et al., 2018), thus locally exciting the postsynaptic neuron for longer. Results indicate that astrocyte-released GABA through GAT-3 acting on the postsynaptic neuron alone is also sufficient to suppress postsynaptic neuronal activity. It was noted that presynaptic neuronal inhibition decreases where astrocytic [Glu] is elevated.

As in Flanagan et al. (2018), consideration was taken of the astrocytic [Glu], reflecting the hypothesized effects of astrocytic glutamine synthetase downregulation (Perez et al., 2012) as observed in the focal sites of some epilepsies, particularly mesial temporal lobe epilepsy (MTLE) (Petroff et al., 2002; Eid et al., 2004). Previously reported results (Flanagan et al., 2018) indicate the slowing of synaptic Glu clearance in line with increasing astrocytic [Glu]. This resulted in the over-activation of postsynaptic NMDA and AMPA receptors and thus heightened local postsynaptic neuronal firing frequencies. Within this paper, the inclusion of astrocytic GAT-3 modulates the postsynaptic firing frequencies despite increased astrocytic Glu content, however, the strength of astrocytic GABA-mediated neuronal inhibition decreases where astrocytic [Glu] is elevated. The implications of this within the context of MTLE would be an ineffective astrocytic GABA-induced synaptic modulation, which would be further impaired by chronic GS downregulation (Hammer et al., 2008), resulting in increased neuronal hyperexcitability and seizure generation.

Although astrocytic-released GABA may act on an extrasynaptic location and the corresponding GABA_A_-Rs have been seen to have a higher affinity to GABA than their synaptic counterparts (Farrant and Nusser, 2005), this has not been accounted for in this model and remains a direction for future work. In addition, this study took account of neuronal inhibition mediated by the ubiquitous GABA_A_-Rs due to experimental results demonstrating their fast activating inhibitory effect of neuronal hyperexcitability and epileptiform activity (Mann et al., 2009). These receptors were modeled to counteract the fast-excitatory behavior of NMDA-R and AMPA-R mediated currents. Further developments of this model would also take account of slower GABA_B_ receptor-activation and their longer-term effects on neuronal hyperexcitability.

## Data Availability Statement

The raw data supporting the conclusions of this article will be made available by the authors, without undue reservation.

## Author Contributions

BF, LM, JW, MT, KW-L, and JH contributed to conception and design of the study. BF wrote the code. BF, LM, and JW wrote the first draft of the manuscript. All authors contributed to manuscript revision, read, and approved the submitted version.

## Conflict of Interest

The authors declare that the research was conducted in the absence of any commercial or financial relationships that could be construed as a potential conflict of interest.
